# Communicating Standing and Walking Data after Spinal Cord Injury: A Patient-Engaged, Qualitative Study

**DOI:** 10.46292/sci23-00019S

**Published:** 2023-11-17

**Authors:** Katherine Chan, Lovisa Cheung, Chris Taylor, Chelsea Wong, Grace Inglis, Kristen Walden, Kristin E. Musselman

**Affiliations:** 1 KITE Research Institute-Toronto Rehabilitation Institute, University Health Network, Toronto, ON, Canada;; 2 Rehabilitation Sciences Institute, University of Toronto, Toronto, ON, Canada;; 3 Department of Physical Therapy, University of Toronto, Toronto, ON, Canada;; 4 Lyndhurst Centre, TRI-UHN, Toronto, ON, Canada;; 5 Praxis Spinal Cord Institute, Vancouver, BC, Canada

**Keywords:** inpatients, interview, rehabilitation, spinal cord injury

## Abstract

**Background:**

The Standing and Walking Assessment Tool has been implemented by physical therapists across Canada, but there is no standardized communication tool to inform inpatients living with spinal cord injury (SCI) about their standing and walking ability.

**Objectives:**

To identify how inpatients with SCI are currently receiving feedback on their standing and walking ability, and to determine if and how they would like to receive information on their standing and walking.

**Methods:**

Ontario's Patient Engagement Framework informed study protocol development. Inpatients with SCI were recruited from a rehabilitation centre in Canada. Purposeful sampling considering severity of SCI and sex was adopted. Three to four months following discharge from inpatient rehabilitation, a semi-structured interview was conducted to explore participants'experiences and preferences regarding feedback on standing and walking ability during inpatient SCI rehabilitation. Interviews were audio-recorded and transcribed verbatim. A conventional content analysis was completed.

**Results:**

Fifteen individuals with SCI (5 female, 10 male) participated. Four themes emerged from the transcripts: (1) motivation for standing and walking, (2) current standing and walking practice, (3) participant preferences for feedback on standing and walking ability, and (4) perceptions of preexisting tools.

**Conclusion:**

Information on standing and walking ability was shared with inpatients with SCI in a variety of ways. Participants identified various preferences for the nature, format, and frequency of feedback concerning standing and walking ability during inpatient rehabilitation, which suggests the need for an individualized approach to communicating this information.

In the first year following a spinal cord injury (SCI), there can be significant change in neurological and motor function,^1^,^2^ often leading to functional changes. For example, the ability to walk may be regained, with the extent of walking recovery influenced by the severity of SCI.^3^ Up to 75% of individuals with motor incomplete SCI (i.e., American Spinal Injury Association Impairment Scale [AIS] grades C and D) regain some walking ability in the first year postinjury.^3^ Documenting change in neurological, motor, and/or functional status is likely useful for all parties involved in SCI rehabilitation—individuals with SCI, clinicians, healthcare institutions, and health systems. Standardized methods to track change may assist with prognostication, treatment planning, discharge planning, resource allocation, and the development of clinical practice guidelines.

Standardized measures of neurological, motor, and/or functional status may also serve as communication tools. They are a means to discuss physical performance and progress with individuals with SCI during their rehabilitation. Clear information and communication, including about the results of treatment, is one of ten quality dimensions used to characterize the patient experience with physical therapy (i.e., the perceived quality of care).^4^ With increasing emphasis on person-centeredness in therapy delivery, the patient experience is increasingly used to measure the quality of health services received.^4^ As a result, organizations have developed tools that facilitate the communication of physical performance and progress with the individuals they serve. For example, the Academy of Neurologic Physical Therapy developed a Physical Therapy Report Card that translates knowledge of performance from the physical therapist to the person with lived experience. The report card provides persons with lived experience with information, in lay terms, about their performance on a core set of standardized outcome measures related to walking, transferring, balance, and balance confidence.^5^

In inpatient SCI rehabilitation in Canada, a standardized approach to the assessment of standing and walking ability has been established through the Standing and Walking Assessment Tool (SWAT) (see **Figure 1**). The SWAT combines stages of walking recovery (i.e., SWAT stages) with established measures of standing and walking, such as the Berg Balance Scale, 10-Meter Walk Test and 6-Minute Walk Test. The SWAT is a valid, reliable, and responsive tool for use with the SCI population^6^-^8^ that has been implemented by physical therapists at rehabilitation hospitals across Canada, but it does not include a communication tool like the Physical Therapy Report Card. Receiving information about performance on the SWAT measures may be of value to individuals with SCI as they participate in rehabilitation. Moreover, this translation of knowledge may contribute to a positive patient experience.

**Figure 1.**
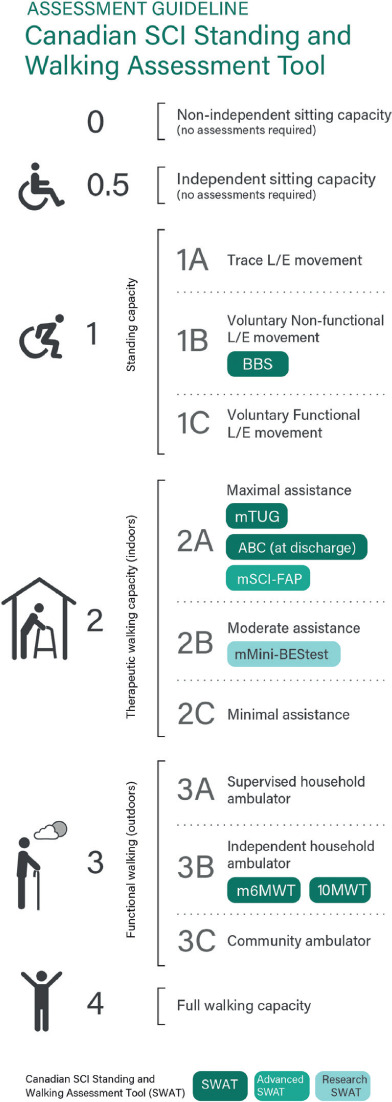
The Standing and Walking Assessment Tool (SWAT). SWAT stages 0-4 (along the left side) identify the measures of standing and walking appropriate for an individual at each stage. A physical therapist would first determine the individual's SWAT stage and then administer the associated standardized measures. Dark boxes indicate the core SWAT measures (i.e., Berg Balance Scale, modified Timed Up and Go, Activities-specific Balance Confidence Scale, modified 6-Minute Walk Test, 10-Meter Walk Test), which are collected across all rehabilitation inpatient hospitals that use the SWAT. Physical therapists have the option to include the modified Spinal Cord Injury-Functional Ambulation Profile (advanced SWAT measure) or the modified Mini-BESTest (research SWAT measure). The stage at which each measure is shown identifies the earliest stage for which the measure is used. (Reproduced with permission from the Canadian SCI Standing and Walking Module Group.)

The objectives of this exploratory, qualitative study were to understand (1) how information about standing and walking ability was translated from physical therapists to inpatients with SCI at a Canadian tertiary rehabilitation hospital and (2) if and how individuals with SCI would like to receive information about their standing and walking ability during inpatient rehabilitation.

## Methods

This descriptive phenomenological^9^ study was conducted at the Lyndhurst Centre in Toronto with ethical approval granted by the University Health Network Research Ethics Board. The design of the study protocol was informed by Ontario's Patient Engagement Framework, which guides the planning, implementation, and evaluation of patient engagement activities.^10^ The framework's guiding principles—partnership, learning, empowerment, transparency, responsiveness, and respect^10^—were interwoven into the study activities to facilitate effective and meaningful engagement. Two engagement approaches were used: (1) consult, in which feedback on a health issue was collected from persons with SCI, and (2) deliberate, in which persons with SCI and health professionals engaged in discussion on a health issue and explored solutions together.^10^ A qualitative research methodology facilitated the generation of detailed insights and allowed participants to be involved in generating solutions; these processes align well with the consulting and deliberating activities of patient engagement.

## Participants

Inpatients with SCI were recruited through the central recruitment service at the Lyndhurst Centre.^11^ Inpatients who met the following criteria were included: (1) diagnosed with a traumatic or nontraumatic SCI and (2) aged ≥18 years. We adopted purposeful sampling,^12^ considering severity of SCI and sex. We aimed to recruit individuals with motor complete SCI and individuals with motor incomplete SCI, enabling us to gain the perspectives of individuals spanning the full spectrum of mobility status. We also aimed to recruit a sample with approximately 60% to 70% males and 30% to 40% females, since SCI is more common among males.^13^-^15^ A moderate sample size of 16 participants was targeted based on the concept of information power (**Table 1**). The information power of our sample was impacted by five key factors (i.e., study aim, sample specificity, established theory, quality of dialogue, analysis strategy); consideration of these factors all together, along a continuum, influenced the sample toward favoring a smaller or a larger sample size. After obtaining written, informed consent, demographic (i.e., age, sex) and injury-related data (i.e., time postinjury, mechanism of injury, neurological level of injury, AIS grade, ambulatory status) were collected by a researcher from the patient chart.

**Table 1.** Determining sample size through information power^27^

Factors of information power	Study details	Favors small sample?
Study aim	Well-defined research questions about a single construct (i.e., feedback on standing and walking ability)	Yes
Sample specificity	Broad inclusion injury-related inclusion criteria	No
Established theory	Planning and analysis guided by Ontario's Patient Engagement Framework^9^	Yes
Quality of dialogue	Experienced qualitative interviewer with expertise in SCI rehabilitation	Yes
Analysis strategy	Cross-case analysis	No

All participants received inpatient rehabilitation at the Lyndhurst Centre in Toronto, which is Canada's largest tertiary rehabilitation hospital for SCI. The Lyndhurst Centre receives, on average, more than 300 inpatient admissions and more than 20,000 outpatient visits each year.^16^ The SWAT is completed with all inpatients at admission to inpatient rehabilitation (i.e., within 7 days of admission), in the week prior to discharge, and at any other time point during their inpatient rehabilitation stay as deemed appropriate by the treating physical therapist.

## Interview

Following discharge from inpatient rehabilitation, a researcher (K.C.) contacted participants monthly to maintain engagement in the study and facilitate the development of trust and rapport.^17^ Approximately 3 to 4 months postdischarge from inpatient rehabilitation, a semi-structured interview was completed by phone. This time point was chosen as participants were able to comment on whether knowledge of their standing and walking performance may have impacted their inpatient experience and their transition to community living.

The interviews were led by either a researcher (L.C.) who had qualitative interviewing experience and was a physical therapist in neurological and musculoskeletal private practice or a physical therapy student (C.T.). As both interviewers had physical therapy backgrounds, they had an in-depth understanding of the neural control and biomechanical requirements of walking, and they understood that regaining the ability to walk was a therapy goal for some individuals living with SCI. At each interview, L.C. and C.T. explained the information produced by the SWAT measures to provide context. L.C. and C.T. then followed a semi-structured interview guide developed by the research team. Participants were asked open-ended questions about (1) what communication, if any, they had with their physical therapist about their standing and walking ability while an inpatient, and (2) what information, if any, they would have liked to receive about their standing and walking ability during inpatient rehabilitation (see **Table 2**). Toward the end of the interview, participants were shown the SWAT (i.e., **Figure 1**) and the Physical Therapy Report Card,^5^,^18^ which includes four core measures of the SWAT. Participants were mailed or emailed the SWAT and report card in advance of the interview and were asked to look at these documents during the interview. The participants’ perspectives on the usefulness of these preexisting tools were gathered. All interviews were audio-recorded.

**Table 2.** Interview guide

Inpatient experience related to standing and walking ability and assessment	Can you tell us about the physical therapy you received during inpatient rehab? Can you tell us about how your standing and/or walking ability was assessed during inpatient rehab? How often was it assessed? Who did the assessments? Was information about your standing and/or walking ability and/or performance on assessments shared with you during inpatient rehab? If yes, how was information regarding standing and/or walking ability shared in inpatient rehab (e.g., written, oral)? How often was this shared? Who shared this information with you? If yes, did you find this information useful? Why or why not? If yes, who did you share this information with, if anyone? If no, do you think that information would have been useful to you? Why or why not? Was it important for you to receive information about your standing and/or walking ability during inpatient rehab? If yes, in what format would you like to receive this information? What are your thoughts about using the SWAT as a method to assess standing and/or walking during inpatient rehabilitation? What information in the SWAT would be meaningful and useful? And, why? If nothing, why? How would you like this information to be shared? [PROBE: verbal, written, etc.] At what point during your rehabilitation would you have liked this information to have been shared? Who would you have liked to share this information with, if anyone? What are your thoughts about using a report card like this to communicate information about standing and/or walking ability during rehabilitation?
Standing and Walking Assessment Tool (SWAT)	
Physical Therapy Report Card	

## Analysis

Demographic and injury-related data were reported descriptively using frequency counts, the mean and standard deviation, or the median and interquartile range, as appropriate. The transcripts of the interviews were the single source of data for this study. The interviews were transcribed verbatim offline by a member of the research team (K.C.). Participants were given the option to review their transcript and edit the content or add information. However, none of the participants opted to receive their transcript. NVivo 12 (QSR International) was used for data management.

A conventional content analysis was completed.^19^ Two researchers (K.C., a kinesiologist with an MSc in Rehabilitation Sciences and qualitative research experience, and C.T., a physical therapy student) independently read two transcripts line-by-line and recorded marginal notes. They then met to compare and discuss the marginal notes and develop codes with definitions (i.e., a preliminary codebook). The preliminary codebook was then applied to another four transcripts by K.C. and C.T. independently, and updates to the codebook were made. The codebook was iteratively applied to and updated for another six transcripts that were independently coded by K.C. and C.T. and reviewed by L.C. The codebook was finalized by K.C. and L.C. after the analysis of the remaining three transcripts. G.I., a person with SCI, and C.W., a physical therapist, reviewed the resulting themes and example quotes. Edits to the themes were made based on their feedback. Their input allowed us to verify our interpretations of the collected data and incorporate their unique perspectives into the analysis.

## Results

Sixteen individuals were enrolled in the study, although one withdrew consent prior to the interview. Fifteen individuals with SCI (5 female, 10 male) aged 59.1 ± 17.3 years participated (**Table 3**). The severity of participants’ injuries was classified as AIS B (*n* = 2), AIS C (*n* = 4), AIS D (*n* = 7), and unknown (*n* = 2).

**Table 3.** Participant demographic and injury-related information

**Participant**	**Sex**	**Age**	**Months postinjury**	**MOI**	**NLI**	**AIS**	**Ambulatory(Y/N)**
P01	F	68	9	Spinal abscess	L4	D	Y
P02	F	80	4	Non-Hodgkin's lymphoma	T1	D	Y
P03	F	63	4	Durotomy	C6	B	N
P04	F	54	9	Lymphoma	T12	D	N
P05	M	69	1	Spinal stenosis with severe cauda equine compression	L2	D	Y
P06	M	78	16	Viral infection	N/A	^	Y
P07	F	59	1	Decompression and cauda equine fusion	L2	C	Y
P08	M	20	3	Cervical decompression	C6	B	N
P09	M	54	2	Epidural correction with cord compression	T7	D	Y
P10	M	70	1	Lumbar stenosis and scoliosis	T11	^	Y
P11	M	38	1	Skeletal metastasis and spinal cord compression	T5	D	Y
P12	M	64	1	Bilateral posterior decompression	C1	D	Y
P13	M	60	3	Vertebral burst fracture	L1	C	Y
P14	M	32	1	Central disk herniation with cauda equine compression	L3	C	Y
P16	M	77	2	Fall	C6	C	N

Note: ^ = AIS unknown; AIS = American Spinal Injury Association Impairment Scale; MOI = mechanism of injury; N/A = not applicable; NLI = neurological level of injury.

At the start of inpatient rehabilitation, nine participants reported their mobility status as nonambulatory. Those who were able to ambulate typically required the use of an assistive device (e.g., walker). Several participants regained their ability to walk with or without an assistive device over the course of inpatient rehabilitation. At the time of the interview, four participants were nonambulatory and 11 participants were able to ambulate with or without an assistive device in their homes, though some participants required the use of a wheelchair when mobilizing in the community.

Four main themes were identified from the interview data: (1) motivation for standing and walking, (2) current standing and walking practice, (3) participant preferences for feedback on standing and walking ability, and (4) perceptions of preexisting tools. **Table 4** outlines the themes, subthemes (if applicable), and example quotes, with Q1, Q2, etc., linking quotes to the text.

**Table 4.** Themes, subthemes, and example quotes

**Theme 1: Motivation for standing and walking**	
Q1: “I am motivated. Whether or not it will lead to the outcome I want, I don't know, but I am motivated.” (P10, ambulatory)	
Q2: “I mean my interest was being able to learn to walk again as normally as possible, to the extent that was possible.” (P09, ambulatory)	
Q3: “I thought some days were not as good as the previous day, but overall like that, I mean [physical therapist] was very encouraging and he told me that he thought I did very well actually like that.” (P16, nonambulatory)	
Q4: “The goal for me was to kind of not use any tool and to kind of independently walk. And that was like the goal for me, even before leaving [rehabilitation center].” (P11, ambulatory)	
Q5: “So, my goals and what I was hoping would be possible in my recovery to get back to those things, being able to kind of, you know walk around and go on hikes, be able to go for a jog, be able to jump theexercise bike or a real bike, and be able to use those things again. So I wanted to to return to the same lifestyle I was living before and to be able to be on my feet, and be able to be moving around.” (P14, ambulatory)	
Q6: “I was a little scared to try the other walker because to me, it didn't feel very sturdy.” (P02, ambulatory)	
Q7: “At home, you can't take chances; if something happens to you, if you fall, there's no one to get you up. Not only that, but if you fall and, let's say you break your rib, well that could be the end so as far as standing and walking goes, at home there is a lot more risk than there was when I was in rehab.” (P12, ambulatory)	
**Theme 2: Current standing and walking assessment practice**	
2a. Assessments conducted during inpatient rehabilitation	Q8: “Walking, standing up and sitting, we did a number of strength tests.” (P14, ambulatory)
	Q9: “Different things, you know, strength test.” (P04, nonambulatory)	
	Q10: “The speed of walking, both comfortable and fast speed…how far I could walk in two and then six minutes durations, a fair amount of standing and balancing exercises, you know, standing with my eyes closed and just standing unaided for a minute or two minutes.” (P06, ambulatory)	
	Q11: “It wasn't really assessed. Like I said, I just used the standing table so it was really just, there wasn't a lot of actual standing done…I was strapped into the table.” (P08, ambulatory)	
	Q12: “That was like maybe three quarters of my, through my stay, like it was near theend.” (P07, ambulatory)	
	Q13: “The only assessments that I got was at the end.” (P16, nonambulatory)	
	Q14: “Yeah they did it at the beginning, yes. And then maybe a month later…they did it again.” (P02, ambulatory)	
	Q15: “Yes absolutely, because I find that the way that he worked is he kind of assessed me day-by-day, which is, which is good because like I was, like at that point, I was just like…the pain was just fluctuating so much.” (P11, ambulatory)	
	Q16: What happened was during my stay, I was there for 50 days, so during my stay half about halfway through, maybe a little bit more than that, my physio, there was a maternity leave. So the one came back so I had to switch physios. And so when the second physio took over, she kind of did the tests again. So that's why I had two of them. Otherwise, I think I would've probably just had the one. But I think the new physio had to do the test again kind of so she could know. (P04, nonambulatory)
2b. Feedback received during inpatient rehabilitation	Q17: “It was just back and forth dialogue, always that dialogue.” (P14, ambulatory)	
	Q18: “I wasn't told anything. Nothing was said to me.” (P12, ambulatory)	
	Q19: “Every two weeks kind of thing.” (P06, ambulatory)	
	Q20: “Every day you went down, there was, you know, different comments made…on your abilities…how you improved.” (P05, ambulatory)	
	Q21: “Every day. Yeah. But you know, that that's when I say that, it's just like asking how you feel, did anything happen, that sort of thing. Not necessarily a hands-on exam or anything.” (P03, nonambulatory)	
	Q22: “So I would have sort of, you know what I would think of as normal conversationswith my wife or my family during visits or on the phone.” (P09, ambulatory)	
	Q23: “Oh just my partner. When I go, we talk about it and all of that.” (P11, ambulatory)	
	Q24: “I also discussed some of that with the physiatrist…and I would see the family doctor during, you know like on a daily basis.” (P09, ambulatory)	
**Theme 3: Participant preferences for feedback on standing and walking ability**	
3a. What the feedback would be useful for	Q25: “Well usually with roommates. Depending on, you know, if it was appropriate to, for them to hear, right? Cause they do have similar problems. So we talked about our doctor's inputs and where we were at.” (P03, nonambulatory)
	Q26: “…after working with them, or talking with them I get an idea what I should be doing…so I can go forward from there.” (P01, ambulatory)	
	Q27: “It could help me in understanding, you know what I can do, what I can't do, what I should be more careful of, et cetera, in my life.” (P09, ambulatory)	
	Q28: “Well it gave me a benchmark to…basically assess my personal, (pause), what'sthe word I'm looking for…growth? As a person who was motivated I found that information very useful.” (P06, ambulatory)	
	Q29: “Of course I found it useful, it was a professional judging me on a measureable scale that, you know an established measureable scale so I was always interested to know exactly where I stood as far as my level of progress was concerned.” (P10, ambulatory)	
	Q30: “…when I get discharged, my, you know, other [physical therapists] that I would be seeing in the community. It would give them a guideline to where I'm at when I'm starting the physiotherapy in the outpatient. And then we could work on the next level, like our goal, my goal and where I'd like to be.” (P07, ambulatory)	
	Q31: “I think overall it's not that important to me.” (P11, ambulatory)	
	Q32: “It doesn't apply to me a whole lot.” (P08, nonambulatory)	
3b. Preferences for format and timing of feedback	Q33: “Orally. And every once in a while, you know, diagrammatically on [the SWAT].” (P10, ambulatory)
	Q34: “Yeah they can just talk to me about it, yes. But he can send an email, that's finetoo.” (P02, ambulatory)	
	Q35: “It would be good, I mean I love the oral, but it would be nice that if there was like sort of like a report card kind of thing. I think written is probably, verbal and written. Because I'd like it explained to me.” (P04, nonambulatory)	
	Q36: “There's so many different things that you're dealing with as an individual when suffering a spinal cord injury that having it communicated to you in more than one way can be useful. And having it on a piece of paper written down, a print off or even available in your electronic portal so you can go back and look on your own time would be useful as well.” (P14, ambulatory)	
	Q37: “I appreciate when I'm working with a physio that they're telling me from one day to the next.” (P04, nonambulatory)	
	Q38: “I would like a monthly update.” (P16, nonambulatory)	
	Q39: “Maybe every month or appropriate time, frequency where you should be improving or if things plateau.” (P03, nonambulatory)	
	Q40: “I would suggest at entry, and part way through and then at completion.” (P06, ambulatory)	
**Theme 4: Perceptions of preexisting tools**	
Q41: “Well, I think the graphics really speak for themselves and that's useful.” (P06, ambulatory)	
Q42: “It's very useful and it's pretty much self-explanatory. [pause] Even if someone is somewhat baffled by numbers and alphabet letters and you know things like ‘trace LE movement’ and ‘voluntary functionalLE movement’…the diagrams to the left of the page are, and the numbers attached to them, are pretty much self-explanatory.” (P10, ambulatory)	
Q43: “Well, at least knowing that you're within normal limits, I think that's very useful.” (P06, ambulatory)	
Q44: “And then what is sort of the average score is also what you want to know cause…you're not expecting to be a marathon runner, but you also want to be, like if this average score is like the norm…Then that works.” (P04, nonambulatory)	
Q45: “So [the report card] tells them and the medical people exactly where you're at and it's a chance for them to say either ‘I'm satisfied’ or ‘I want to go forward’.” (P03, nonambulatory)	
Q46: “[The SWAT] specifically measures over a period of time someone's progress…whether or not someone has plateaued, whether or not someone is declining.” (P10, ambulatory)	
Q47: “[The Physical Therapy Report Card] sounds like it's a nice, simple way to show whether there's improvement or no improvement.” (P07, ambulatory)	
Q48: “Cause then at least you see growth [with the report card]...with kind of a date.” (P04, nonambulatory)	
Q49: “[The SWAT] presents some…goals that I can work towards.” (P03, nonambulatory)	
Q50: “[The SWAT] would be helpful because then you can set your goal to the next level. You know what the next level is and you'd have some sort of a goal to keep going, it gives you like a guidance.” (P07, ambulatory)	
Q51: “Yeah, the only way I can kind of think of it is if somebody came up to me and said, ‘You're a 1A, whichmeans you can do this’. I would go, ‘Oh yes, I know I can do that’ whereas this is, you actually get to realize like some of you here, how far you can walk in meters, how fast you walk in meters/second. Like that's a lot more useful information, the fact that I'm a 1A doesn't matter to me. How far I can actually, I'm able to do these things fast, I'm able to do these things, would be a lot more useful to me.” (P08, nonambulatory)	
Q52: “When you ask me if I've improved, well yesterday I couldn't walk to the door, but today I can, you know what I mean. Like so how do I put a number on that?” (P05, ambulatory)	
Q53: “But for the most part, I just find that like [the report card is] a lot of information for like a regular person to even need. To be honest with you. Yeah. Like I think there, at a certain point it's important to kind of like know where your status is. But at the same time like I don't think I need to know this all the time.” (P11, ambulatory)	
Q54: “I feel like just my status changes all the time. Like even when I was inpatient as well. So like there there's no report card that could, you know keep up with that. It was so crazy in terms of like how my pain fluctuated and like how that may, that changes everything, right?” (P11, ambulatory)	

### 1. Motivation for standing and walking

Participants indicated that having a motivation to improve their standing and walking ability led to an interest in receiving feedback about their performance during inpatient rehabilitation. Two-thirds of participants (*n* = 10) expressed having motivation to improve their standing and walking ability (Q1, Q2), with this motivation beginning early after injury. Participants mentioned receiving encouragement from their physical therapist (Q3), which contributed to their motivation. They also discussed specific goals for their walking ability, such as walking without a gait aid (Q4) and participating in recreational activities they enjoyed prior to injury (Q5). Two participants further explained that their motivation to improve standing and walking ability was influenced by concerns about the quality of their walking movements, their safety when trying a new gait aid (Q6), and the possibility of falling in the home environment (Q7).

### 2. Current standing and walking assessment practice

Participants outlined how information pertaining to their standing and walking ability was obtained and shared in inpatient physical therapy, resulting in two subthemes: (a) assessments conducted during inpatient rehabilitation, and (b) feedback received during inpatient rehabilitation.

#### 2a. Assessments conducted during inpatient rehabilitation.

Participants explained that assessment of standing and walking ability during inpatient rehabilitation was mainly implemented by physical therapists; however, the implementation varied according to ambulatory status and timing within their inpatient hospital stay. In additional to physical therapists, therapy assistants and an occupational therapist were also noted to occasionally perform these assessments. Participants recalled tests of balance, range of motion, strength, walking, and advanced walking tasks (e.g., avoiding obstacles) being administered (Q8-Q10). Nonambulatory (*n* = 2) participants reported a lack of assessment of their standing and walking (Q11). This lack of assessment for nonambulatory participants was the only between-group difference identified during the secondary comparison analysis.

The timing of assessments varied from participant to participant. Some participants mentioned being assessed once (i.e., at admission, midpoint, or before discharge) (Q12, Q13), while other participants were assessed at least twice during the course of their inpatient rehabilitation (Q14) or on a day-today basis (Q15). One participant stated that they were assessed during a transition of care (Q16).

#### 2b. Feedback received during inpatient rehabilitation.

Most participants reported receiving feedback about their standing and walking ability during inpatient rehabilitation; however, the format and frequency of that feedback varied. Feedback on standing and walking ability was received in various formats: verbal, written, electronic, and hybrid (i.e., combination of two or more formats), with verbal feedback being most common (Q17). One participant (P04, nonambulatory) “took a picture of the copy” so they would have the option of sharing the results with their outpatient physical therapist. In contrast, three participants, one of whom was nonambulatory, stated that they did not receive any feedback (Q18).

For the participants who did receive feedback, they recalled feedback being received biweekly (Q19) or daily (Q20). However, the daily feedback was not necessarily directly related to their standing and walking ability (Q21). Upon receiving feedback on their standing and walking ability, participants shared that information with family and friends (Q22, Q23) and other healthcare professionals (Q24) in their circle of care. Two participants, including P03 (nonambulatory), mentioned sharing feedback with their hospital roommate, as this was someone also experiencing SCI (Q25).

### 3. Participant preferences for feedback on standing and walking ability

Participants’ preferences for receiving feedback on their standing and walking ability during inpatient rehabilitation are reflected in two subthemes: (a) what the feedback would be useful for, and (b) preferences for format and timing of feedback.

#### 3a. What the feedback would be useful for.

Most participants (*n* = 13) stated that receiving feedback on their standing and walking ability would be useful for goal setting (Q26), gaining insight into their physical abilities (Q27), and monitoring progression of their therapy (Q28, Q29). It was also noted that the feedback could be shared with the next physical therapist that a participant would work with after hospital discharge (Q30). Contrarily, two participants, one of whom was nonambulatory, stated it was not important for them to receive feedback on their standing and walking ability (Q31, Q32).

#### 3b. Preferences for format and timing of feedback.

Participants preferences for how feedback on their standing and walking ability is received varied; however, the majority (*n* = 10) preferred a hybrid format with oral input combined with written or visual information (Q33-Q35). Having a written or electronic copy of the feedback would enable individuals to review the information whenever most appropriate for them during the early postinjury phase (Q36). Two participants indicated they preferred daily feedback on their standing and walking ability (Q37), while others (*n* = 9) preferred to receive feedback at intermittent time points, such as monthly (Q38, Q39) or at specific time points during their hospital stay (Q40).

### 4. Perceptions of preexisting tools

After the SWAT and Physical Therapy Report Card were introduced and explained to the participants, they provided their thoughts and opinions of each tool. Overall, participants indicated that both tools were clear and could be helpful when setting goals, expectations, and monitoring progress, especially if the information was linked to functional changes. The SWAT stages were described as “comprehensive” (P03, nonambulatory), “self-explanatory” (P07, ambulatory), “quite clear” (P11, ambulatory), and “straight forward” (P16, nonambulatory). At least seven participants found that the diagrams were helpful to understand the SWAT (Q41, Q42). Similarly, the Physical Therapy Report Card was described as “pretty self-explanatory” (P08, nonambulatory) and “straight-forward” (P14, ambulatory). Providing a range of average scores from community-dwelling adults was perceived to be a useful aspect of the report card (Q43, Q44). Participants indicated that both tools could display their current standing and walking status (Q45) and progression through inpatient rehabilitation (Q46-Q48). It was also suggested that the SWAT stages could facilitate goal setting related to standing and walking ability (Q49, Q50).

Despite these potential benefits, some participants (*n* = 3) mentioned that numbers and scores were not as important as tangible, functional changes in their standing and walking ability (Q51, Q52). As stated by P13 (ambulatory), “I don't go by papers, I go from my performance… how my body talks to me.” It was also suggested that the amount of information provided on the report card was more than necessary for a person with SCI (Q53) and that the report card would not be able to reflect the constant changes in their function (Q54).

## Discussion

This exploratory, descriptive qualitative study described how information about standing and walking ability was translated from physical therapists to inpatients with SCI at a Canadian tertiary rehabilitation hospital and identified patient preferences for the receipt of feedback about their standing and walking ability. The majority of participants described having motivation and goals to improve their ability to stand and/or walk. There is considerable variety in the nature, format, and frequency of feedback on standing and walking ability that inpatients with SCI receive. Verbal feedback is most common, possibly because written records are created for clinicians and the patient's chart. Participants indicated that feedback is useful for goal setting, monitoring therapy progress, and communicating their abilities to other healthcare professionals, families, and peers. Verbal feedback combined with written or visual information was the preferred format for receiving feedback. Preexisting tools, such as the SWAT figure and Physical Therapy Report Card, may facilitate the feedback process, but some participants stressed the value of seeing tangible, functional changes in their standing and walking ability rather than focusing on scores on clinical assessments.

Taken altogether, the findings suggest most individuals with SCI are interested in receiving feedback on their standing and walking ability during inpatient rehabilitation; however, a key finding was the variability in the desired format (e.g., verbal, written), frequency (e.g., daily, monthly), and nature (e.g., functional achievements, scores on clinical measures) of the feedback. This finding suggests a personalized approach is needed. At the beginning of inpatient rehabilitation, the physical therapist and individual with SCI could jointly create a feedback plan that reflects the individual's preferred format, frequency, and nature of feedback. The plan could be revisited throughout the individual's hospital stay as preferences may change as recovery progresses. Explicitly personalizing the feedback process through consideration of the individual's needs and preferences aligns with the philosophy of person-centered care.^20^ Individuals with SCI want to contribute more to the decision-making of their care, and their involvement facilitates empowerment, feeling capable, and increased autonomy— all desired outcomes of the rehabilitation process.^21^

To facilitate the exchange of personalized feedback on standing and walking ability, it would be helpful to have a variety of feedback tools that clinicians can select from. The Physical Therapy Report Card is one such tool. Participants in the current study indicated that the SWAT figure may be a useful feedback tool, albeit with some modifications as the SWAT figure was not designed for this purpose. Other tools may exist that could be modified to share feedback on standing and walking. For example, inpatients receiving medical care have expressed interest in a progress bar with important steps and past/future events related to their care displayed; a similar tool could be developed for standing and walking rehabilitation.^22^ The collection of a variety of resources available to clinicians may have the benefit of reducing their workload, as standardized tools would likely take less time to complete than a nonstandardized form or tool. By creating a variety of feedback tools, a toolkit on standing and walking feedback could be developed. Toolkits are a collection of varied knowledge translation products that collate explicit knowledge.^23^ They are a low-cost strategy to facilitate clinical implementation, and their use has been linked to improved quality of care for other clinical populations.^24^-^26^

We identified three limitations of our study. First, the interviews were conducted over the phone due to COVID-19 restrictions, so we were unable to observe participants’ body language and nonverbal cues. Second, only four participants were nonambulatory at the time of the interview; hence, the study findings primarily reflect ambulatory individuals with SCI. Third, we only collected the perspectives of inpatients with SCI, but not the clinicians who provide feedback on standing and walking ability. Future work could focus on probing clinician perspectives on sharing feedback on standing and walking ability with their patients, especially as tools for providing feedback are developed.

Feedback on standing and walking ability provided to inpatients with SCI should be individualized according to the preferences and needs of each individual with SCI. The nature, format, and frequency should be jointly determined by the individual living with SCI and their physical therapist. The creation and implementation of feedback tools may facilitate the delivery of personalized feedback on standing and walking ability after SCI.
